# Antihypertensive medication versus health promotion for improving metabolic syndrome in preventing cardiovascular events: a success rate-oriented simulation study

**DOI:** 10.1186/1472-6947-11-8

**Published:** 2011-02-13

**Authors:** Yoichi Ohno, Satomi Shibazaki, Ryuichiro Araki, Takashi Miyazaki, Mayuko Hanyu, Makiko Satoh, Tsuneo Takenaka, Hirokazu Okada, Hiromichi Suzuki

**Affiliations:** 1 Community Health Science Center, Saitama Medical University, Saitama, Japan; 2 Department of Nephrology, Saitama Medical University, Saitama, Japan

## Abstract

**Background:**

In practice, it is difficult to compare the effectiveness of traditional antihypertensive treatment with that of health promotion in reducing incidence rate of cardiovascular disease (IR_CVD_, events/year). This simulation study compared the effectiveness of two approaches to reducing IR_CVD _in a sample population: a traditional approach, in which high-risk patients are treated with conventional antihypertensive medications, and a population-based approach, in which subjects participate in a health promotion program.

**Methods:**

We constructed a simulation model for a sample population of middle-aged Japanese men whose systolic blood pressure (SBP) levels are normally distributed (130 ± 20 mm Hg). The principal assumption was that IR_CVD _increases exponentially according to SBP. The population IR_CVD _was calculated as the product of the distribution of SBP multiplied by IR_CVD _at each SBP. The cumulative IR_CVD _was calculated by the definite integral from the lowest to the highest SBP of IR_CVD _at each SBP level. The success rates were calculated according to SBP and metabolic risk profiles in the two approaches, respectively.

**Results:**

The reduction in IR_CVD _was twice as large for antihypertensive medications as it was for health promotion in several situations. For example, if adherence to antihypertensive treatment occurred at a realistic level, the decrease in IR_CVD _was estimated at 9.99 × 10^-4^. In contrast, even if the health program was promoted optimistically, the decrease in IR_CVD _was estimated at 4.69 × 10^-4^.

**Conclusions:**

The success rate-oriented simulation suggests that prescribing antihypertensive medications is superior to promoting the health promotion program in reducing IR_CVD _in virtual middle-aged Japanese men.

## Background

Metabolic syndrome is a significant risk factor for the development of cardiovascular disease (CVD) in the Japanese middle-aged population [1,2]. The Japanese Ministry of Health, Labour and Welfare has recently forced health insurance organizations to provide opportunities for their members to take annual health check and health promotion programs to prevent and recover from metabolic syndrome. Some epidemiologists who support the government health and welfare policy insist that a nationwide population-based approach has a greater impact on the prevention of CVDs than does a traditional approach in which antihypertensive medications are prescribed to high-risk patients (http://www.niph.go.jp/soshiki/jinzai/koroshoshiryo/tokutei/) [3,4]. People who satisfy the Japanese criteria for metabolic syndrome are advised to take the health promotion program to decrease the effects of metabolic syndrome by losing weight or reducing their waist circumference. Public health nurses, with the assistance of dietitians, exercise instructors, and occasionally regional primary physicians and industrial physicians, supervise the supporting dietary treatment and physical exercise of the program. The guidelines of the health promotion recommend patients with both metabolic syndrome and hypertension to first consult their primary physician and, after reducing their blood pressure (BP) to less than 160/100 mm Hg, to take the health promotion program separately [5,6]. In contrast, the Japanese Society of Hypertension (JSH) 2009 hypertension treatment guidelines insist that hypertensive subjects (BP > 140/90 mm Hg) with metabolic syndrome should visit primary physicians before initiating the health promotion. As the consequence of merging the two guidelines, the algorithm is too complicated to be familiar to primary physicians and members of health insurance organizations. Moreover, in practice it is difficult to begin the two approaches concurrently because most individuals are willing to accept only one lifestyle change at a time [7]. Therefore, our concern is which should come first: traditional antihypertensive treatment, including established lifestyle education, or vigorous lifestyle interventions. Direct evidence is still lacking with regard to the beneficial effects of lifestyle interventions (except for smoking cessation) on cardiovascular events [8,9].

Epidemiological studies take several years and incur huge costs to draw conclusions about whether the health promotion program could substitute for traditional antihypertensive medications in reducing cardiovascular events. In a situation in which obvious clinical evidence is unavailable and extremely difficult, primary physicians have to decide which procedure is suitable for an individual patient with metabolic syndrome with hypertension: the traditional antihypertensive treatment or the health promotion program [3]. Simulation studies may offer patients with hypertensive metabolic syndrome and their primary physicians a possible course of action until clinical evidence becomes available [10-12]. We aimed in this simulation study to assess whether the Japanese health promotion program for improving metabolic syndrome has a large impact on the treatment of metabolic syndrome with hypertension. Should primary physicians postpone their prescriptions for antihypertensive medications, even if their patients' BP levels are higher than 140/90 mm Hg? We calculated the anticipated reduction in the incidence rate of CVD (IR_CVD_, events/year) that would follow from conventional antihypertensive treatment and from the newly introduced health promotion program in a model population from the viewpoint of BP regulation.

## Methods

### Calculation of the IR_CVD _in a sample population

To simplify the simulation, we set a hypothetical sample population. Systolic BP (SBP = *x*) was assumed to be normally distributed and to represent the sum of the risk factors for cardiovascular complications. In other words, other risk factors, including metabolic profiles, were transferred onto the SBP level. The predictive value of metabolic syndrome for CVD is considerably determined by that level [13,14]. The contribution of metabolic factors to CVD is estimated as being much smaller in Japan than in Western countries [15] because the incidence of coronary heart disease in Japan is approximately 1/3 of that in Western countries [5]. In the model population, the mean (μ) and the standard deviation (σ) of the SBP levels were hypothesized to be 130 and 20 mm Hg, respectively, which were consistent with those of Japanese male subjects at 60 years of age [16]. The probability density function equals [17]

$$f(x)=\frac{1}{\sqrt{2\pi }\sigma }\exp\left(-\frac{{\left(x-\mu \right)}^{2}}{2\sigma^{2}}\right)=\frac{1}{\sqrt{2\pi }\times 20}\exp\left(-\frac{{\left(x-130\right)}^{2}}{2\times 20^{2}}\right).$$

Earlier epidemiological data suggested that the slope of the curve on the scattergram for the relationship between SBP and IR_CVD _was exponential [5]. IR_CVD _was assumed by the following equation and based on the hypertension guidelines and epidemiological data [16,18,19]: IR_CVD _= $P(x)=\frac{1}{l}\exp\left(\frac{x-m}{k}\right)$, where k, l, and m are constants and represent the slope of the exponential curves, the absolute value of IR_CVD_, and the rising point from e^0 ^= 1, and where simulations were performed when k = 20, 30, or 40; l = 100; and m = 140. The particular numbers of the constants were adopted to be consistent with epidemiological data in Japan [5,16]. In this situation, the cumulative IR_CVD _of the sample population is 0.01 events/year, which corresponds to a moderate risk for CVD or that of middle-aged Japanese men. These constants are influenced by risk factors such as smoking and glucose and lipid profiles. Subjects with diabetes or previous CVD are out of our scope because CVD risk assessment is different from our estimation [5]. Effects of other medications such as statin and aspirin are estimated independently from those of antihypertensive medications [20].

**Figure 1:**
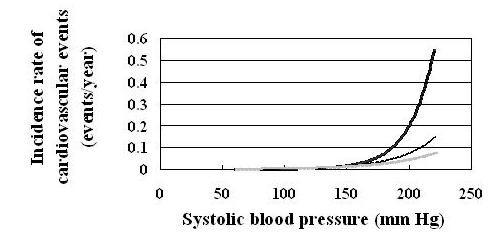
**Relationship between systolic blood pressure (SBP) and cardiovascular event rate (IR_CVD_)**. IR_CVD _=$\frac{1}{l}\exp\left(\frac{SBP-m}{k}\right)$. The thick black, thin black, and thick gray lines represent k = 20, 30, and 40, respectively, where l = 100 and m = 140. For example, when k = 20, the IR_CVD _is 0.0036/year, 0.01/year, and approximately 0.027/year when SBP is 120, 140, and 160 mm Hg, respectively, and corresponds to a moderate risk in the Japanese Society of Hypertension 2009 guidelines.

The cumulative IR_CVD _was calculated by the definite integral from the lowest to the highest SBP of the IR_CVD _at each SBP level in the sample population [3]. The population IR_CVD _was evaluated as the product of distribution according to SBP multiplied by the cardiovascular event rate at each SBP (Figure 1).

The cumulative IR_CVD_

$$\begin{array}{l} =\int_{L}^{H}\left\{f(x)\times P\left(x\right)\right\}dx\\ =\int_{L}^{H}\left\{\frac{1}{\sqrt{2\pi }\times 20}\times \exp\left(-\frac{{\left(x-130\right)}^{2}}{2\times 20^{2}}\right)\times \frac{1}{l}\exp\left(\frac{x-m}{k}\right)\right\}dx, \end{array}$$

Where *f*(*x*) and *P*(*x*) represent probability density function and IR_CVD _according to SBP, respectively. When k = 20, l = 100, m = 140, and

$$\begin{array}{l} \frac{x-130}{20}=y,\\ \int_{L}^{H}f(x)\times P\left(x\right)dx=\int_{L}^{H}\left\{\frac{1}{\sqrt{2\pi }}\times \exp\left(-\frac{y^{2}}{2}\right)\times \frac{\exp\left(y-\frac{1}{2}\right)}{100}\right\}dy\\ \approx \int_{-\infty }^{\infty }\frac{1}{\sqrt{2\pi }}\times \exp\left(-\frac{{\left(y-1\right)}^{2}}{2}\right)\times \frac{1}{100}dy=0.0\text{1} \end{array}$$

### IR_CVD _after administration of antihypertensive medications

In this simulation study, we assumed that IR_CVD _after the interventions would gradually converge with IR_CVD _directly calculated from the SBP levels after the interventions. Similar to the estimates used in earlier simulation studies [3,21], CVD risk estimates after interventions were calculated by using a formula before interventions that was based on the SBP level after interventions. Risk reduction in cardiovascular complications by antihypertensive treatment is expected to reach ~50% within several years [18]. Therefore, we estimated that a 50% of the decrease in SBP by the two approaches would contribute to IR_CVD _reduction in several years.

IR_CVD _after administration of antihypertensive medications

$$\begin{array}{l} =\int_{L}^{H}\left\{f_{1}(x)\times P_{1}\left(x\right)\right\}dx\\ =\int_{L}^{e}\left\{f_{0}\left(x\right)\times P_{0}\left(x\right)\right\}dx+\int_{e}^{H}\left\{f_{0}\left(x\right)\times P_{0}\left(x-g\left(x\right)\right)\times s\left(x\right)\right\}dx+\int_{e}^{H}\left\{f_{0}\left(x\right)\times P_{0}\left(x\right)\times \left(1-s\left(x\right)\right)\right\}dx, \end{array}$$

Where *e*, *g*(*x*), and *s*(*x*) represent the SBP level at the initial administration of antihypertensive medications, the SBP-lowering effect of the antihypertensive medications and success rate of antihypertensive treatment. Antihypertensive medications were applied to hypertensive patients in the sample with an SBP > 140, 150, and 160 mm Hg. The consultation rate for medical care was estimated as a function of SBP, and the SBP-lowering effects of the antihypertensive medications were simulated in several situations. In this simulation study, beyond-BP effects that are expected in some antihypertensive medications and in the non-pharmacological treatment of the usual clinical settings were assumed to be factored into the SBP-reducing values. Beyond-BP effects in some antihypertensive medications were considered to be relatively small compared with BP lowering in preventing CVD [20]. We adopted stepwise combination medications to control SBP to the target level, if necessary, as the Japanese hypertension treatment guidelines recommend, predominantly with an angiotensin II receptor blocker and a calcium channel blocker [22].

**Figure 2:**
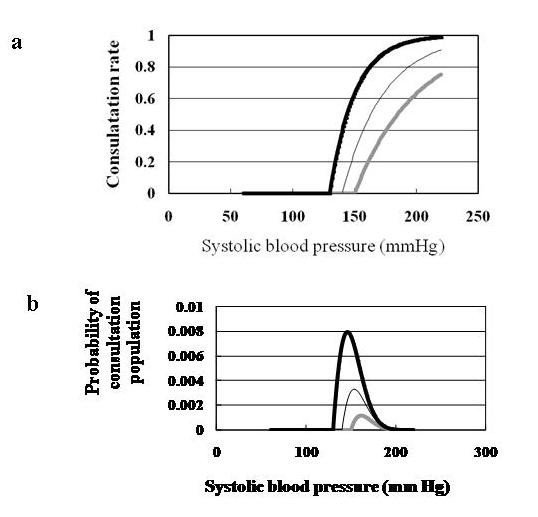
**Relationship between systolic blood pressure (SBP) and consultations**. (a) SBP and primary physician consultation rate of hypertensive patients. The consultation rate = 1-exp(-(SBP-a)×b) (a = alarming SBP level, above which patients should be concerned; b = the accessibility factor to a primary physician). The thick black (a = 130, b = 0.05), thin black (a = 140, b = 0.03), and thick gray (a = 150, b = 0.02) lines represent ideal, optimistic, and realistic situations, respectively. (b) SBP and probability of consultation with a primary physician. The probability equals the product of the probability density in the sample population and the consultation rate.

Antihypertensive treatment constitutes at least two of the following major components: the consultation rate with primary physicians after patients are diagnosed with hypertension at the annual health check and the achievement rate in lowering BP to the target SBP level. However, few reports are available concerning these components [23], especially in Japan [24]. Moreover, the physician consultation rate for medical care after patients are diagnosed with hypertension in an annual health check program is seldom investigated in regional settings. The Japanese government mandates primary physicians to report the number of patients with certain common diseases on 1 day every 3 years. The consultation rates are estimated from the ratio of the total number of outpatients who visited medical facilities on the patient survey day to the total population of Japan (http://www.mhlw.go.jp/toukei/saikin/hw/kanja/05/02-02.html). Given these circumstances, we proposed a few assumptive equations to calculate IR_CVD _on the basis of primary healthcare settings and earlier epidemiological investigations [18,25]. We adopted an exponential mathematical formula to estimate the consultation rate and the success rate in order to produce a feasible calculation in combining the normal distributions of SBP levels, considering that the consultation rate should get closer to 100% if SBP raises [26]. First, the consultation rate of primary physicians after the annual health check program for metabolic syndrome is assumed to be represented by the following equation: consultation rate (patients who visited primary physicians/patients who are diagnosed with hypertension in the annual health check) = 1-exp(-(SBP-a)*b) (a = an alarming SBP of 130, 140, and 150 mm Hg, above which patients should be concerned about their BP; and b = an accessibility factor to a primary physician of 0.05, 0.03, and 0.02) (Figure 2a). The consultation rate has a large impact on the size of the population (Figure 2b). The particular numbers of the constants were adjusted to realistic Japanese situations (approximately 20-30%) [5,24]. Conversely, the rate without consulting a medical doctor = exp(- (SBP - a) * b). Second, the SBP-lowering effect of antihypertensive medication is assumed by the following equation: SBP-lowering effect = (SBP-c)*d (c = the target SBP level of 130, 140, and 150 mm Hg; d = adherence for reaching the target SBP level of 0.6, 0.7, and 0.8) [22]. A primary physician's compliance with hypertension treatment guidelines [23] and a patient's drug adherence constitute important parts of factor "d" [27]. Antihypertensive medication is simulated as being initiated from the following SBP levels: e = 140, 150, and 160 mm Hg [28]. Earlier studies demonstrated varying levels of physician adherence to hypertension guidelines, which were usually disappointingly low. To cite a case of hypertensive patients whose SBP is 160 mm Hg, when k = 20, l = 100, and m = 140, the IR_CVD _before intervention is approximately 0.027/year (Figure 1); with an optimistic situation of a = 140 and b = 0.03, a consultation rate of 0.45 (Figure 2a), and a realistic SBP-lowering effect (c = 140, d = 0.7, and e = 150, or [160-140] × 0.7 × 0.5 = 7 mm Hg), the IR_CVD _after antihypertensive medications is approximately 0.019/year.

### IR_CVD _after health promotion

IR_CVD _after heath promotion

$$\begin{array}{l} =\int_{L}^{H}\left\{f_{1}(x)\times P_{1}\left(x\right)\right\}dx\\ =\int_{L}^{H}\left\{f_{0}(x)\times P_{0}\left(x-\alpha \right)\right\}dx\times \beta +\int_{L}^{H}\left\{f_{0}\left(x\right)\times P_{0}\left(x\right)\right\}dx\times \left(1-\beta \right), \end{array}$$

Where *f*_0_(*x*) and *P*_0_(*x*) represent the probability density function and IR_CVD _of cardiovascular events before intervention, respectively, and *f*_1_(*x*) and *P*_1_(*x*) represent the probability density function and IR_CVD _of cardiovascular events after intervention, respectively. *α *and *β *represent the SBP-lowering effect, including the other cardiovascular risks and the success rate of health promotion programs, respectively. We simulated the health promotion program for metabolic syndrome as being applied to the total population. The effects of the specific health promotion program on cardiovascular complications are assumed to be 5, 10, and 20 mm Hg when they are represented as SBP-lowering effects in several years. The PREMIER trial has recently reported that lifestyle modifications for BP control (sodium reduction, weight loss, and increased physical activity) decreased 10 mm Hg in SBP and 0.0002 events/year reduction in heart attack rate by in middle-aged, healthy, untreated individuals with prehypertension or stage I hypertension [9]. A pilot non-randomized controlled study in Japan has recently indicated that a 27-month community-based lifestyle approach to CVD resulted in 1 mm Hg and 3 mm Hg differences in SBP between the intervention and control groups for men and women, respectively [29].

The health promotion program has three support systems, stratified according to metabolic risk profiles: (1) an affirmative support system to improve the effects of metabolic syndrome continuously for 6 months (more than three close contacts with health care nurses), (2) a support system to motivate metabolic syndrome candidates to improve their health conditions (two contacts with health care nurses), and (3) a support system to offer individual information for health promotion when the results of the annual health check are sent. In the simulation, we calculated the success rates of these strategies at three levels: (1) realistic, (2) optimistic, and (3) ideal. The calculations in each scenario are summarized in Table 1. The Japanese Ministry of Health, Labour and Welfare has advocated a success rate of 10% by the end of fiscal year 2013 (http://www.niph.go.jp/soshiki/jinzai/koroshoshiryo/tokutei/). To cite a case of hypertensive patients whose SBP is 160 mm Hg, when we promote the health program optimistically (α = 10 mm Hg), the IR_CVD _after health promotion at the corresponding SBP level (160 - 10 × 0.5 = 155 mmHg) is 0.021. The success rate is estimated as β = 0.275 in the optimistic situation.

**Table 1:** Success rate of health promotion programs

	Support system to improve metabolic syndrome			Indifferent to metabolic syndrome	Σ
	Affirmative campaign	Motivating campaign	Offering information		
Realistic pattern	10	20	50	20	13.75
	37.5	25	10	0	

Optimistic pattern	10	20	50	20	27.5
	75	50	20	0	

Ideal pattern	10	20	50	20	50
	100	75	50	0	

The upper and lower numbers in each cell represent the proportion of candidates assigned to the corresponding program and its success rate. Σ in the right-most column represents the total success rate, calculated as the sum of the products of the proportion of candidates assigned to the program multiplied by its success rate.

The comparison between the conventional BP-lowering treatment and the health promotion program for improving metabolic syndrome is based on the following two indexes: the reductions in IR_CVD _by the two approaches and the number of health checks (N_HC_) needed to decrease one cardiovascular complication per year in each approach.

$${\text{N}}_{\text{HC}}=\frac{1}{\int_{L}^{H}f_{0}\left(x\right)\times P_{0}\left(x\right)dx-\int_{L}^{H}f_{1}\left(x\right)\times P_{1}\left(x\right)dx},$$

Where *f*_0_(*x*) and *P*_0_(*x*) represent the probability density function and IR_CVD _before intervention, respectively, and *f*_1_(*x*) and *P*_1_(*x*) represent the probability density function and IR_CVD _after intervention, respectively. After confirming the accuracy of the results by comparing the authentic definite integral method and the numerical analytical approach, we performed these calculations by using numerical analysis with 1 mm Hg intervals.

## Results

**Figure 3:**
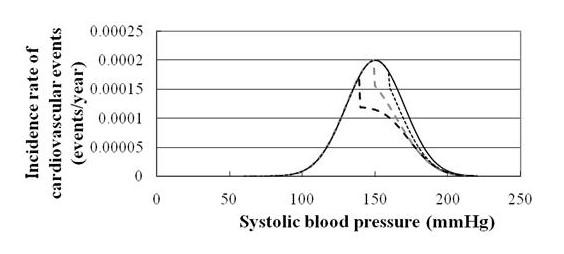
**Relationship between systolic blood pressure and cardiovascular event rate before and after prescribing traditional antihypertensive medications**. The solid black, dotted black (>160 mm Hg), dashed grey (>150 mm Hg), and dashed black (>140 mm Hg) lines represent no antihypertensive treatment and realistic, optimistic, and ideal antihypertensive treatments, respectively. The area between the solid black line and the corresponding line represents the reduction in the incidence rate of cardiovascular events.

The cumulative risk reduction with traditional antihypertensive medications is demonstrated in the representative combinations of several factors, such as a, b, c, d, and e, when IR_CVD _is assumed to be an exponential function, or $P(x)=\frac{1}{l}\times \exp\left(\frac{x-m}{k}\right)$ (Table 2). Figure 3 demonstrates that the relationship between SBP and IR_CVD _is reduced by prescribing antihypertensive medications in virtual situations.

**Table 2:** Reduction in cardiovascular event rate by traditional antihypertensive medications in a hypothetical population

k = 20	a = 130 mm Hg	a = 140 mm Hg	a = 150 mm Hg	a = 160 mm Hg
	b = 0.05	b = 0.03	b = 0.02	b = 0.01
	c = 130 mm Hg	c = 140 mm Hg	c = 150 mm Hg	c = 160 mm Hg
	d = 0.8	d = 0.7	d = 0.6	d = 0.5
e = 140 mm Hg	2.33 × 10^-3^			
e = 150 mm Hg	2.08 × 10^-3^	**9.99 × 10**^**-4**^		
e = 160 mm Hg	1.56 × 10^-3^	**8.46 × 10**^**-4**^	**4.52 × 10**^**-4**^	
e = 180 mm Hg	4.51 × 10^-4^	3.02 × 10^-4^	1.92 × 10^-4^	6.64 × 10^-5^

k = 30	a = 130 mm Hg	a = 140 mm Hg	A = 150 mm Hg	a = 160 mm Hg
	b = 0.05	b = 0.03	b = 0.02	b = 0.01
	c = 130 mm Hg	c = 140 mm Hg	c = 150 mm Hg	c = 160 mm Hg
	d = 0.8	d = 0.7	d = 0.6	d = 0.5

e = 140	1.13 × 10^-3^			
e = 150	9.68 × 10^-4^	**4.24 × 10**^**-4**^		
e = 160	6.74 × 10^-4^	**3.42 × 10**^**-4**^	**1.76 × 10**^**-4**^	
e = 180	1.58 × 10^-4^	1.01 × 10^-4^	6.26 × 10^-5^	2.06 × 10^-5^

k = 40	a = 130 mm Hg	a = 140 mm Hg	A = 150 mm Hg	a = 160 mm Hg
	b = 0.05	b = 0.03	b = 0.02	b = 0.01
	c = 130	c = 140	c = 150	c = 160
	d = 0.8	d = 0.7	d = 0.6	d = 0.5

e = 140	7.32 × 10^-4^			
e = 150	6.14 × 10^-4^	**2.58 × 10**^**-4**^		
e = 160	4.11 × 10^-4^	**2.02 × 10**^**-4**^	**1.02 × 10**^**-4**^	
e = 180	8.66 × 10^-5^	5.40 × 10^-5^	3.31 × 10^-5^	1.06 × 10^-5^

Abbreviations: k = constant determining the slope of the exponential curve between systolic blood pressure and cardiovascular incidence rate; l = constant related to absolute value of cardiovascular incidence rate of 100; m = constant determining the slope of the exponential curve to be e^0^/l of 140; a = an alarming SBP of 130, 140, 150, and 160 mm Hg, above which patients should be concerned about their BP; b = an accessibility factor to a primary physician of 0.05, 0.03, 0.02, and 0.01; c = the target SBP level of 130, 140, 150, and 160 mm Hg; d = adherence for reaching the target SBP level of 0.5, 0.6, 0.7, and 0.8; e = SBP level of 140, 150, 160, or 180 mm Hg from which administration of antihypertensive medications was initiated.

**Figure 4:**
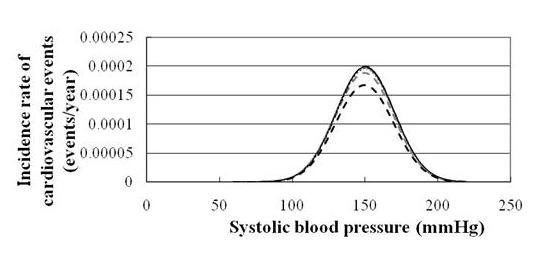
**Relationship between systolic blood pressure and cardiovascular event rate before and after promoting health promotion programs**. The solid black, dotted grey, dashed black, and dashed gray lines represent no health promotion program and realistic, optimistic, and ideal health promotion programs, respectively. The area between the solid black line and the corresponding line represents the reduction in cardiovascular event rates. The solid black line overlaps the dotted grey line considerably.

The cumulative risk reduction accompanied by the health promotion program is calculated in the representative combinations of several factors, such as *α *and *β *(Table 3 and Figure 4).

**Table 3:** Reduction in incidence rate of cardiovascular events per year by the health promotion program in a hypothetical population

k = 20	α = 20 mm Hg	α = 10 mm Hg	α = 5 mm Hg
β ideal	1.35 × 10^-3^	8.52 × 10^-4^	4.92 × 10^-4^
β optimistic	7.42 × 10^-4^	**4.69 × 10**^**-4**^	**2.71 × 10**^**-4**^
β realistic	3.71 × 10^-4^	**2.34 × 10**^**-4**^	**1.35 × 10**^**-4**^

k = 30	α = 20 mm Hg	α = 10 mm Hg	α = 5 mm Hg

β ideal	8.40 × 10^-4^	5.05 × 10^-4^	2.90 × 10^-4^
β optimistic	4.62 × 10^-4^	**2.78 × 10**^**-4**^	**1.59 × 10**^**-4**^
β realistic	2.31 × 10^-4^	**1.39 × 10**^**-4**^	**7.97 × 10**^**-5**^

k = 40	α = 20 mm Hg	α = 10 mm Hg	α = 5 mm Hg

β ideal	6.31 × 10^-4^	3.72 × 10^-4^	2.16 × 10^-4^
β optimistic	3.47 × 10^-4^	**2.05 × 10**^**-4**^	**1.19 × 10**^**-4**^
β realistic	1.74 × 10^-4^	**1.02 × 10**^**-4**^	**5.93 × 10**^**-5**^

Abbreviations: k = constant determining the slope of the exponential curve between systolic blood pressure and cardiovascular incidence rate; l = constant related to absolute value of cardiovascular incidence rate of 100; m = constant determining the slope of the exponential curve to be e^0^/l of 140; *α *= SBP-lowering effect (mm Hg), including the other cardiovascular risks; *β *= success rate of health promotion program.

The N_HC _to rescue one cardiovascular event per year is much lower for antihypertensive medications than it is for health promotions in corresponding virtual situations. For example, in the case of k = 20, l = 100, and m = 140, if we prescribe antihypertensive medicines to patients whose SBP is not less than 150, accessibility to the medical office is 0.03 or the usual situation, the target SBP is set to 140 mm Hg, and the success rate is 0.7, ΔIR_CVD _and N_HC _are estimated at 9.99 × 10^-4 ^and 1001, respectively. In contrast, even if we can promote the health program optimistically, participants reduce their body weight (BW) by ~5 kg, and their risk reduction is estimated at a 10 mm Hg SBP-lowering effect, IR_CVD _and N_HC _are estimated at 4.69 × 10^-4 ^and 2134, respectively.

In Figure 3 and Figure 4, ΔIR_CVD _is shown as the subtracted area of the IR_CVD _before and after the two approaches. Below the SBP level at which antihypertensive medications were prescribed,ΔIR_CVD _at each SBP level is slightly higher in Figure 4 than in Figure 3. However, above the SBP level at which antihypertensive medications were prescribed, ΔIR_CVD _at each SBP level is much higher in Figure 3 compared with the corresponding simulation curves.

## Discussion

Our success rate-oriented simulation demonstrates that antihypertensive medications are more effective than the health promotion program for improving metabolic syndrome in reducing the risk of cardiovascular events. This result suggests that primary physicians should treat hypertensive patients whose SBP levels are not less than 140 mm Hg with medications, as the traditional hypertensive guidelines recommend. Although this simulation allows a substantial role for the health promotion program in reducing cardiovascular events, risk reduction with traditional antihypertensive medications is superior. The novelty of our simulation is in the estimation of success rates for both approaches, although we used several ambitious assumptions. The consultation rate has a large impact on reducing cardiovascular events through the prescription of antihypertensive medications.

The population-based approach to the prevention of cardiovascular events by improving metabolic syndrome may be more unsatisfactory than expected for several reasons: (1) The risk reduction of health promotion for improving metabolic syndrome in CVD might be underestimated. We took the BP-lowering effect of health promotion to improve metabolic syndrome into account for reducing cardiovascular complications. The BP-lowering effect by reducing BW is estimated at 1 to 2 mm Hg/kg BW loss [5,25,30]. The effect of the health promotion program on lowering SBP is limited, and the effect on reducing risk might be beyond what we anticipated [25,31-33]. The health promotion program can reduce the risk of cardiovascular complications through pathways without lowering BP. For example, weight loss improves metabolic factors such as glucose and lipid profiles [8,34], and physical activity can inhibit the production of reactive oxygen species [35]. In addition, the beneficial effects of the interaction between lowering BP and metabolic factors in inhibiting cardiovascular events may go beyond what we anticipated. (2) The risk of cardiovascular events increases exponentially with SBP elevation. For this assumption, the sum of the risk of cardiovascular complications at SBP levels of less than 140 mm Hg is small even when the slope of the curve for the relationship between SBP and IR_CVD _is low. In other words, BP is an excellent predictor of CVD, especially in Japan, where stroke has been more prevalent than ischemic heart disease. Hypertension is a determinant component in Japanese men with metabolic syndrome for predicting cardiovascular complications [36].

One criticism of our model is that the estimation of both approaches may be too optimistic. Only a few researchers have reported the consultation rate in a general Japanese population [24].

Another assumption of our model relates to adherence of the primary physician to the hypertensive treatment guidelines and the patients' adherence to medication. The success rate of antihypertensive treatment, according to the Japanese antihypertensive treatment guidelines, is reported to be 30 to 60% [37,38]. We assumed that IR_CVD _after the interventions will gradually converge with IR_CVD _directly calculated from the SBP levels after the interventions, as shown in another simulation [21]. This estimation is based on the guidelines and the evidence derived from large-scale clinical investigations and long-term follow-up studies [5,18,39].

One question about our model concerns what would happen if traditional antihypertensive medications and the health promotion program were administered concurrently. If the effects of both approaches are independent, then the overall effect of both approaches is the difference between the sum and the product of each approach. However, this assumption is too robust to adopt because most hypertensive patients with metabolic syndrome might be encouraged to lose weight in the medical office of the outpatient department, the usual clinical setting, not as part of a health promotion approach offered by a health nurse. In addition, the success rate of the health promotion program will decrease if patients are satisfied with the reduction in SBP as a result of taking antihypertensive medications. Most individuals are willing to accept only one approach at a time [7]. These factors greatly interfere with the independency of both approaches.

Another question relates to why we boldly assumed the transfer of metabolic risk factors onto SBP levels. There are few reports available that address how to transfer metabolic risk factor onto SBP levels. The estimation of the relationship between the SBP levels and IR_CVD _is a key factor in assessing these issues. In the simulation model, the absolute value of IR_CVD _related to factor "l" does not influence the results of the simulation. A sensitive question is whether traditional antihypertensive medications are more effective if we restricted the subjects whose SBP levels are within the high normal range (130-139 mm Hg) from the model. The superiority of the medication would become unclear in the high normal SBP range (data not shown).

Many undefined issues in preventive medicine emerge from this pioneer simulation study. In future, the consultation rate and medication adherence in various situations should be investigated vigorously. This study indicates that the consultation rate has a tremendous impact on IR_CVD _in a high-risk approach. We propose that two components are related to the consultation rate: awareness of hypertension and accessibility to medical care. However, many other factors and relations such as cost-effectiveness, economic status, and preventive health information need to be explored.

## Conclusions

A traditional approach of using antihypertensive treatment is superior to the health promotion approach for improving metabolic syndrome by reducing the risk of cardiovascular events in a sample population of middle-aged Japanese men. However, the health promotion approach also has a substantial impact on reducing cardiovascular events if success rate is satisfactory in this group.

## Competing interests

The authors declare that they have no competing interests.

## Authors' contributions

YO did all aspects of this research. SS, RA, and TM wrote the epidemiological part of the draft. MH and MS analyzed the effect of the population approach. TT, HO, and HS analyzed the effect of the high-risk approach. All authors read and approved the final manuscript.

## Pre-publication history

The pre-publication history for this paper can be accessed here:

http://www.biomedcentral.com/1472-6947/11/8/prepub
